# ^18^F-FDOPA PET/CT for neuroendocrine tumors in multiple endocrine neoplasia type 1

**DOI:** 10.3389/fendo.2026.1849746

**Published:** 2026-08-05

**Authors:** Aisha A. Tepede, Maya Lee, James Welch, Adel Mandl, Craig Cochran, Ahmed Hamimi, Lee S. Weinstein, William F. Simonds, Ahmed M. Gharib, Corina Millo, Samira M. Sadowski, Jenny E. Blau, Smita Jha

**Affiliations:** 1 Metabolic Diseases Branch, National Institute of Diabetes and Digestive and Kidney Diseases (NIDDK), National Institutes of Health, Bethesda, MD, United States; 2 Biomedical and Metabolic Imaging Branch/National Institute of Diabetes and Digestive and Kidney Diseases (NIDDK), National Institutes of Health, Bethesda, MD, United States; 3 Clinical Center PET Department (CC PET), National Institutes of Health, Bethesda, MD, United States; 4 Surgical Oncology Program, National Cancer Institute (NCI), National Institutes of Health, Bethesda, MD, United States

**Keywords:** functional neuroendocrine tumors, hepatic metastases, insulinomas, pancreatic neuroendocrine tumors, Zollinger-Ellison syndrome

## Abstract

**Objective:**

The aim of this study was to evaluate the efficacy of 6-18F-fluoro-L-3,4-dihydroxyphenylalanine (^18^F-FDOPA) positron emission tomography/computed tomography (PET/CT) in the identification of primary and metastatic NETs in patients with multiple endocrine neoplasia type 1 (MEN1).

**Design:**

This prospective MEN1 cohort study evaluated 16 genetically confirmed patients with MEN1. Inpatient subjects were imaged using CT, magnetic resonance imaging (MRI), ^68^Ga-DOTATATE PET/CT, and ^18^F-FDOPA PET/CT. A composite reference test combining up to 3 years of historical imaging and a multidisciplinary evaluation was used against the index test. Lesion-, patient-, and site-based analyses were performed.

**Setting:**

The NIH Clinical Center was the setting for this study.

**Patients:**

Patients with germline *MEN1* variants confirmed to have MEN1 were included in the study.

**Results:**

A total of 134 lesions were identified after review of CT, MRI, and ^68^Ga-DOTATATE PET/CT. ^18^F-FDOPA PET/CT detected 17% (23/134, 95% CI: 8.2–27.1). Of the total study participants, 50% (8/16) of the patients had ^18^F-FDOPA PET/CT uptake in primary and metastatic sites with overlapping ^68^Ga-DOTATATE PET/CT uptake and localization on CT and MRI. Region-based analysis showed that ^18^F-FDOPA PET/CT had the highest avidity for lung (4/12, 33%) and pancreatic (8/38, 21%) NETs.

**Conclusion:**

^18^F-FDOPA PET/CT has low efficacy overall in detecting MEN1-related NETs.

**Clinical Trial Registration:**

https://clinicalstudies.info.nih.gov/protocoldetails.aspx?id=000344-DK&&query=, identifier NCT04969926.

## Introduction

Multiple endocrine neoplasia type 1 (MEN1) is a rare autosomal dominant syndrome characterized by multiple, often simultaneous primary neuroendocrine tumors (NETs) predominantly in the anterior pituitary, parathyroid, duodenum, or the pancreas. Gastroenteropancreatic NETs (GEP-NETs) account for ~70% of deaths in patients with MEN1 (1). Patients undergo lifelong imaging surveillance to monitor the growth of these NETs and to evaluate new lesions and/or metastases. Imaging plays a central role in the diagnosis and management of duodenopancreatic NETs (dpNETs) in MEN1 as, currently, no available laboratory tests provide optimal surveillance.

Currently available imaging modalities for NETs include conventional cross-sectional anatomic imaging with multiphasic (dual-phase arterial and portal venous) contrast-enhanced computed tomography (CT) or magnetic resonance imaging (MRI), as well as endoscopic ultrasound (EUS). Functional imaging has evolved from ^111^In-pentetreotide scintigraphy, which detected only 31% of NET lesions, to somatostatin receptor (SSTR)-positron emission tomography (PET) using ^68^Ga-DOTATATE (or ^68^Ga-DOTATOC, ^64^Cu-DOTATATE), which demonstrates 95% sensitivity for GEP-NETs and is now approved by the Food and Drug Administration (FDA) and widely utilized for the detection, staging, and assessment of SSTR expression to guide peptide receptor radionuclide therapy (PRRT) eligibility. However, imaging sensitivity remains limited by tumor size, location, background tissue density, tumor heterogeneity, and variable SSTR expression. MRI demonstrates superior diagnostic accuracy compared to CT for pancreatic NETs in patients with MEN1, with positive predictive values of 91%–100% versus 60%–100% for CT, and offers the critical advantage of avoiding ionizing radiation in patients requiring lifelong surveillance (2). SSTR-PET/CT detects significantly more lesions than conventional imaging (95% vs. 45% for anatomic imaging alone) but must be interpreted in combination with dual-phase CT or MRI to identify both SSTR-positive and SSTR-negative disease, as approximately 2%–8% of well-differentiated GEP-NETs demonstrate absent, weak, or heterogeneous SSTR expression (3, 4). Notably, two-thirds of insulinomas are SSTR-PET negative, and poorly differentiated grade 3 neuroendocrine carcinomas typically lack SSTR expression, requiring alternative imaging strategies such as ^18^F-FDG PET/CT (5). Current consensus guidelines recommend MRI as the preferred surveillance modality for MEN1-related dpNETs due to its superior accuracy and lack of radiation exposure, with SSTR-PET/CT and EUS reserved for specific clinical scenarios where results will impact management decisions, such as detecting occult metastases in tumors >10 mm, comprehensive presurgical staging, or determining PRRT eligibility (6).

Another tracer, 6-18F-fluoro-L-3,4-dihydroxyphenylalanine (^18^F-FDOPA) has emerged as a useful tool in the clinical management of some NETs, particularly functional NETs (7–9). Lesion detection by ^18^F-FDOPA PET/CT is reliant on the expression of L-type amino acid transporters (LAT1 and LAT2) on the surface of NETs. Little is known about the efficacy of ^18^F-FDOPA PET/CT for the detection of primary and metastatic NETs in patients with MEN1 syndrome. The purpose of this study was to determine the accuracy of ^18^F-FDOPA PET/CT in detecting neuroendocrine neoplasms in MEN1.

## Methods

### Patients

**Figure 1:**
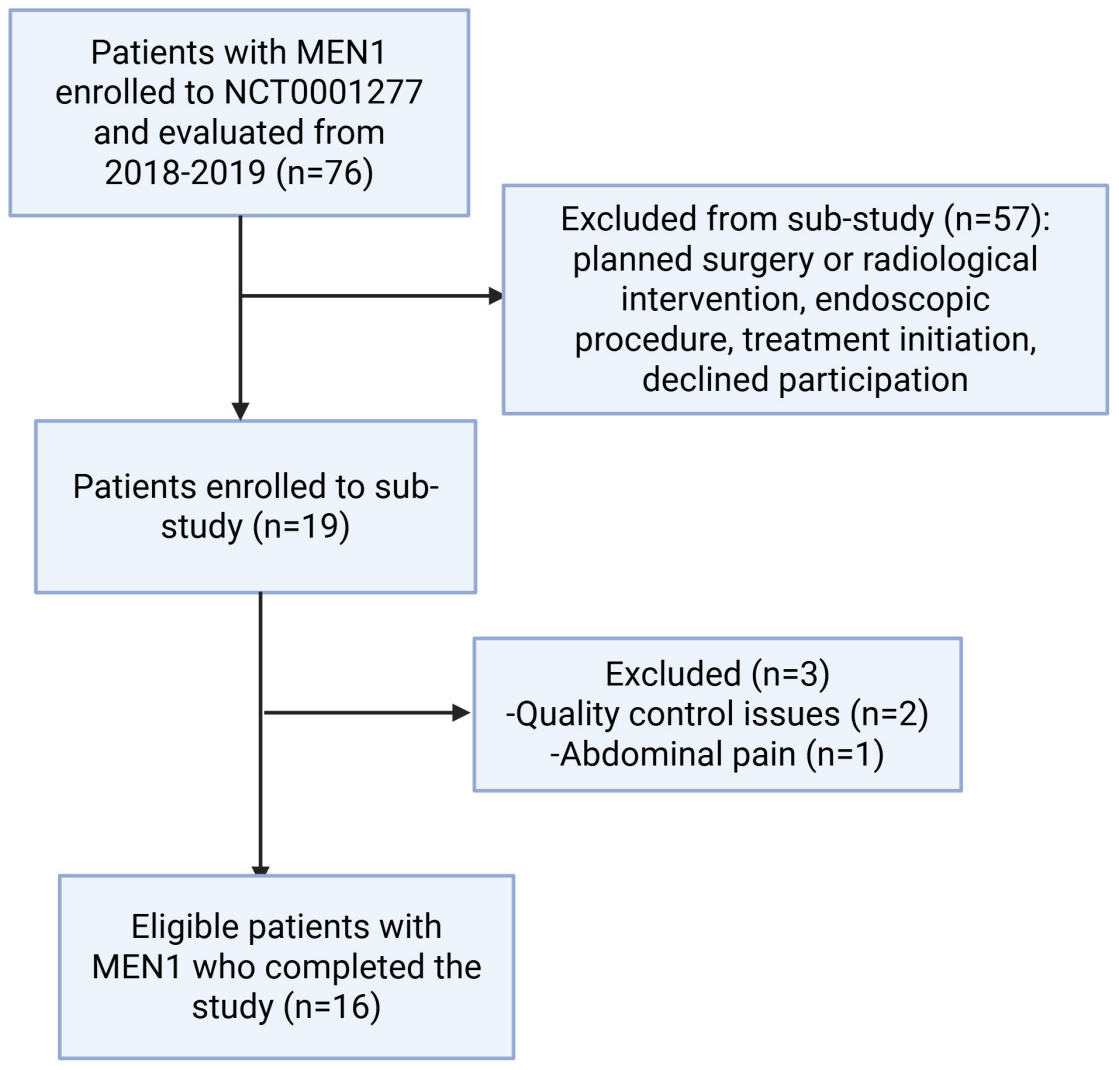
A study flowchart describing the patient cohort.

Patients were eligible to participate if they were 18 years or older and were already part of a long-standing IRB-approved natural history protocol for MEN1 (NCT0001277). Eligible patients were those with genetically confirmed MEN1 undergoing routine follow-up at our institution (**Figure 1**). Patients requiring specific testing for surgical workup and those undergoing surgery or procedures such as esophagogastroduodenoscopy or interventional radiology were excluded due to procedure prioritization and scheduling. Imaging studies included ^68^Ga-DOTATATE PET/CT and ^18^F-FDOPA PET/CT, in addition to standard-of-care images (CT and MRI as described). Patients were also excluded if they were unable or unwilling to give informed consent, had serious underlying medical conditions that restricted diagnostic testing, were pregnant (tested by a urine pregnancy test) or lactating, had a concurrent active infection, had a known hypersensitivity to carbidopa, or were concurrently taking a nonselective monoamine oxidase (MAO) inhibitor. Written informed consent was obtained from all study participants. As part of the surveillance, patients underwent a history and physical exam, routine biochemical testing, and imaging studies including CT and MRI of the chest, abdomen, and pelvis.

### Biochemical evaluation

Routine biochemical evaluations included but were not limited to ionized calcium, gastrin, glucose, insulin-like growth factor-1 (IGF-1), prolactin, thyroid-stimulating hormone (TSH), parathyroid hormone (PTH), sodium, creatinine, chromogranin A, alanine aminotransferase (ALT), aspartate aminotransferase (AST), and free thyroxine (FT4). Twenty-four-hour urinary studies included calcium, creatinine, and 5-hydroxyindoleacetic acid (5-HIAA).

### Imaging evaluation

For ^18^F-FDOPA PET/CT scans, carbidopa (200 mg) was given 60 min before intravenous injection with 444 MBq mCi of ^18^F-FDOPA. For ^68^Ga-DOTATATE PET/CT, as per previous studies at our center (3), each patient was injected intravenously with 185 MBq ^68^Ga-DOTATATE. Patients were fasted for at least 4 h prior to the PET examination with water permitted. Patients were positioned supine in a PET/CT scanner, and images were obtained from the top of the skull to the upper thighs. A low-dose, non-contrast-enhanced CT was used for attenuation correction and anatomic localization. Maximum standardized uptake values (SUVmax) were measured based on patient total body weight for each functional imaging.

CT scans of the neck, chest, abdomen, and pelvis were performed using LightSpeed Ultra and LightSpeed QX/i scanners (General Electric Healthcare Technologies, Waukesha, WI) as well as an Mx8000 IDT scanner (Philips Medical Systems, Andover, MA) with the following settings: non-contrast, arterial and portal venous phase, 2-mm slices, with rapid infusion of non-ionic, water-soluble contrast agent (130 mL injected at 2 mL/s), and oral contrast. Chest, abdominal, and pelvic MRI were performed with slices at a thickness of 6 mm to include T2 series with and without fat saturation and short tau inversion recovery and T1 pre- and postcontrast series, after intravenous injection of gadoliniumdiethylentriamine pentaacetic acid (Siemens Verio 1.5 Tesla; Siemens Medical Solution, Malvern, PA; and Philips Achieva 1.5 and 3 Tesla; Philips Medical Systems).

### Data analysis

^18^F-FDOPA PET/CT and ^68^Ga-DOTATATE PET/CT are frequently used as clinical and research imaging modalities at our institution. For this study, the nuclear medicine study physician was masked to the results of other imaging examinations and to the extension of tumor metastasis in study patients. Lesion was considered abnormal for both ^18^F-FDOPA PET/CT and ^68^Ga-DOTATATE PET/CT when it showed uptake clearly higher than non-tumor background and not attributable to normal physiological activity. Data collected for identified lesions on nuclear imaging included location and SUVmax.

CT and MRI are known to have varying sensitivities and specificities in NET detection among different organs in MEN1 (10). Given the importance of evaluating different anatomic imaging modalities for the detection of NETs in this research study, all known primary or metastatic MEN1 chest and abdomen organ lesions (*primary*: lung, stomach, pancreas, duodenum, and adrenal; *metastatic*: liver, lymph nodes, and bone; *other*: pituitary and parathyroid were evaluated separately) were included (based on imaging characteristics of NETs) regardless of discrepancies between CT and MRI. Pure cystic lesions were not considered NET and therefore not included. Pituitary, parathyroid, and bone findings were included in a descriptive analysis as they were not the focus of this analysis.

Additionally, for this study, the preferred reference standard (histopathology) is neither feasible nor ethical because none of the patients required surgical intervention. Challenges for pathological confirmation in this population requires use of similar methodologies applied in familial neuroendocrine syndromes (11).

### Lesion adjudication workflow

We employed a structured lesion adjudication process as described below. All lesion adjudication was performed during multidisciplinary tumor board review by a panel comprising radiologists, nuclear medicine physicians, endocrinologists, endocrine surgeons, and oncologists. Adjudication followed a hierarchical, stepwise process designed to minimize reliance on any single imaging modality:

Step 1: Anatomic imaging anchor. Lesion candidacy was first established on the basis of anatomic imaging (CT and/or MRI). A lesion was considered a candidate NET if it demonstrated solid or partially solid morphology with imaging characteristics consistent with a NET (e.g., arterial-phase hyperenhancement for pancreatic lesions, enhancing hepatic lesions with characteristic wash-out pattern, and solid pulmonary nodules). Pure cystic lesions were excluded at this stage. Anatomic imaging served as the primary anchor for lesion identification, ensuring that the reference standard was not solely dependent on functional imaging.

Step 2: Functional imaging concordance. Candidate lesions identified on anatomic imaging were then cross-referenced with ^68^Ga-DOTATATE PET/CT. Lesions demonstrating both anatomic imaging characteristics of NET and concordant DOTATATE uptake (focal uptake exceeding non-tumor background, not attributable to physiological biodistribution) were classified as confirmed NETs with high confidence.

Step 3: Longitudinal confirmation. For lesions with discordant findings between anatomic and functional imaging (i.e., anatomically suspicious but DOTATATE-negative, or DOTATATE-positive without a clear anatomic correlate), longitudinal imaging data obtained over a period of up to 3 years were reviewed. A lesion demonstrating interval growth or stability on serial anatomic imaging (CT and/or MRI) in a patient with genetically confirmed MEN1 was classified as a confirmed NET based on temporal behavior, independent of functional tracer uptake. In case of lesions without an anatomic correlate, lesional uptake seen on two successive scans at least 6 months apart was classified as confirmed. Since study participants had all been followed at our center for several years prior to their participation in this study, longitudinal scans were available for all participants. Any lesion that remained discordant at this point was assessed based on future follow-up imaging with CT/MRI and ^68^Ga-DOTATATE PET/CT obtained under the natural history protocol since the completion of ^18^F-FDOPA PET/CT.

Lesion-based analysis: The number of lesions per patient identified by the composite reference was compared to those identified by ^18^F-FDOPA PET/CT using paired Wilcoxon signed-rank test.

Patient-based analysis: Patient-level detection rate was calculated as the proportion of patients with at least one lesion detected on ^18^F-FDOPA PET/CT relative to the composite reference.

Region-based analysis: A site or region was considered positive if at least one lesion was detected in that site based on composite reference. The denominator of the site detection rate was the number of patients in whom that site was positive based on composite reference. Site-based detection rates were compared between the ^18^F-FDOPA PET/CT and the composite reference by McNemar’s test.

Standard errors and 95% confidence intervals of proportion of positive lesions were estimated from 2,000 bootstrap samples by random sampling with replacement on the participant level to account for intra-patient correlation arising from multiple lesions. The 2.5th and 97.5th percentiles of 2,000 bootstrap samples were taken as the 95% confidence limits. Spearman rank correlation was used to measure and test the association between biochemical variables of interest and number of lesions per patient. All tests were two-sided and *p*-values <0.05 were considered statistically significant.

## Results

A total of 19 patients were enrolled and 16 patients completed the study. Two patients were unable to obtain ^18^F-FDOPA PET/CT due to tracer quality control issues. In addition, one patient developed abdominal pain during the ^68^Ga-DOTATATE PET/CT and therefore the scan was aborted (**Figure 1**). The mean age was 55 (range: 30–81) years, and 38% (6/16) were female. MEN1-related characteristics and baseline biochemical parameters are presented in **Table 1**. A total of 134 lesions were identified among 16 patients using all imaging modalities: ^68^Ga-DOTATATE, CT, and MRI (**Table 2**).

**Table 1:** Baseline information on study participants.

Characteristic	Mean ± SD	Normal range
Patient total	16	
Female, *n* (%)	6 (38%)	
Age (range)	55 (30–81)	
*MEN1* variant type		
Frameshift	10	
Missense	2	
Nonsense	1	
Deletion	2	
Splice Site	1	
Hyperparathyroidism diagnosis, *n* (%)	16 (100%)	
PTH level (pg/mL)	97.9 ± 91.6	15.0–65.0
Ionized calcium (mmol/L)	1.3 ± 0.12	1.12–1.32
Pituitary adenoma diagnosis, *n* (%)	9 (56%)	
Functional adenoma	2/16 (13%)	
Prolactin level (μg/L)	12.1 ± 6.7	2.0–25.0
Pancreatic neuroendocrine tumor diagnosis, *n* (%) *	14 (87.5%)	
Insulinoma	1/16 (6%)	2.6–24.9
Zollinger–Ellison diagnosis, *n* (%)	12 (75%)	
Gastrin (pg/mL)	1,299 ± 3,701.9 (>12× ULN)	<100
Lung NET diagnosis, *n* (%)	8 (50%)	
Previous abdominal surgeries, *n* (%)	13 (81.3%)	
Metastatic NETs (n)	13 (81.3%)	
Only local/regional metastasis	3/16 (19%)	
Liver metastasis ± local/regional	10/16 (63%)	
5-HIAA (mg/24 h; range)	6.6 ± 4.9 (2.2–22)	≤8.0
Chromogranin A level (ng/mL; range)	1,224 ± 1,447.1 [(>12×ULN); 81–5,323]	<93

*One patient had total pancreatectomy; ULN, upper limit of normal.

**Table 2:** Lesions detected by ^18^F-FDOPA PET/CT by location.

Lesion by location	No. of lesions detected by FDOPA (%)	95% CI
Total	23/134 (17)	8.2–27.1
Lung	4/12 (33)	0–71.4
Gastric	1/7 (14)	0–60
Pancreas	8/38 (21)	5.1–38.9
Duodenum	1/11 (9)	0–42.9
Liver	2/28 (7)	0–19.1
Adrenal	1/6 (17)	0–50
Lymph node	6/32 (19)	6.7–33.3

### Functional imaging:

^18^F-FDOPA PET/CT detected 17% of lesions (23/134; 95% CI: 8.2%–27.1%) among 8/16 patients (50%) with a mean SUVmax of 12.2 ± 14.8. Except for the adrenal lesion, all lesions detected on ^18^F-FDOPA PET/CT also had uptake on ^68^Ga-DOTATATE PET/CT.

### Lesion analysis:

Lung NETs: A total of 12 lung lesions were identified in eight patients. ^18^F-FDOPA PET/CT identified four lesions among three patients with a mean SUVmax of 4.4 ± 0.77 (range: 3.6–5.1).

Gastric/Duodenal NETs: At study entry, 12/16 patients (75%) carried the diagnosis of Zollinger–Ellison syndrome. Seven gastric lesions were identified among five patients. Of these, ^18^F-FDOPA PET/CT localized one gastric lesion (SUVmax: 30) that was not seen on CT/MRI but showed positive uptake on ^68^Ga-DOTATATE PET/CT. In the duodenum, a total of 11 lesions were identified in seven patients. Only one of these lesions was seen on ^18^F-FDOPA PET/CT, which was not anatomically identified on CT/MRI (SUVmax: 31).

Pancreas: All 16 patients had a prior diagnosis of pancreatic neuroendocrine tumors (pNETs). One patient had undergone total pancreatectomy, and another had no evidence of residual pNET at the time of surveillance. Overall, ^18^F-FDOPA PET/CT identified 21.1% (8/38; 95% CI: 5.1%–38.9%) of lesions with a mean SUVmax of 8.7 ± 4.3.

Adrenal: CT/MRI identified six adrenal lesions across six patients with a mean lesion size of 1.6 ± 0.57 cm (range: 0.9–2.8 cm). ^68^Ga-DOTATATE PET/CT detected 50% of lesions (3/6) with a mean SUVmax of 31.2 ± 4.6. ^18^F-FDOPA PET/CT identified one adrenal lesion (0.9 cm) with a low SUVmax of 1.99, which was not seen on ^68^Ga-DOTATATE PET/CT. In this patient (DK-1188), blood and urinary tests of adrenal function were normal as described previously (12). This tumor has since been resected and was histologically confirmed to be pheochromocytoma.

**Figure 2:**
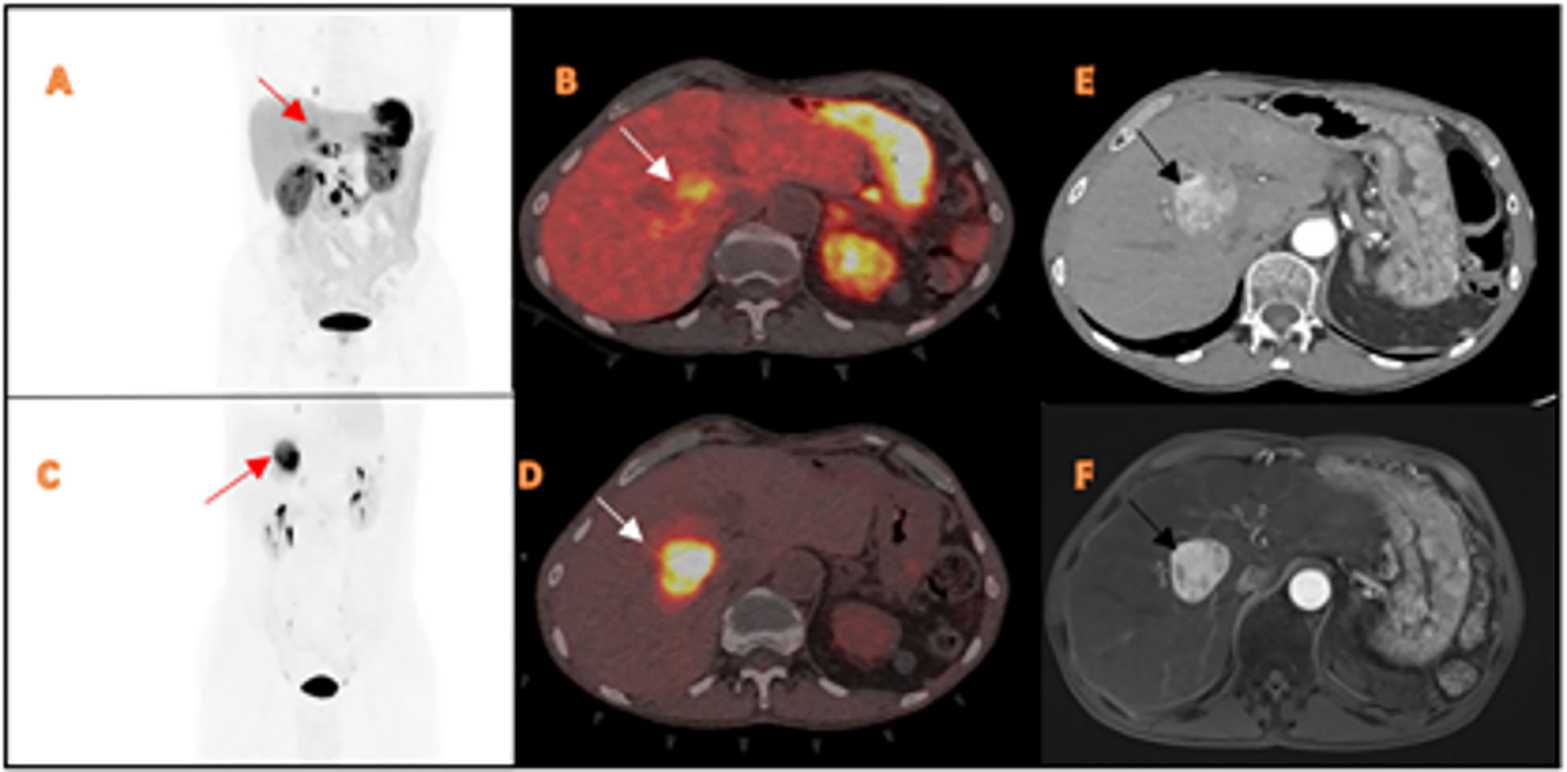
A 66-year-old man with MEN1 (DK-59) complicated by metastatic Zollinger–Ellison syndrome and gastric carcinoids. **(A, B)** ^68^Ga-DOTATATE PET/CT demonstrates limited/partial lesion uptake in the liver on maximum intensity projection (red arrow) and axial view (white arrow); SUV maximum 14.0. **(C, D)** ^18^F-FDOPA PET/CT demonstrates increased uptake of the liver metastases on maximum intensity projection (red arrow) and axial view (white arrow); SUV maximum 45.0. **(E, F)** CT and MRI show corresponding liver metastases measuring 3.0 cm (black arrow).

**Figure 3:**
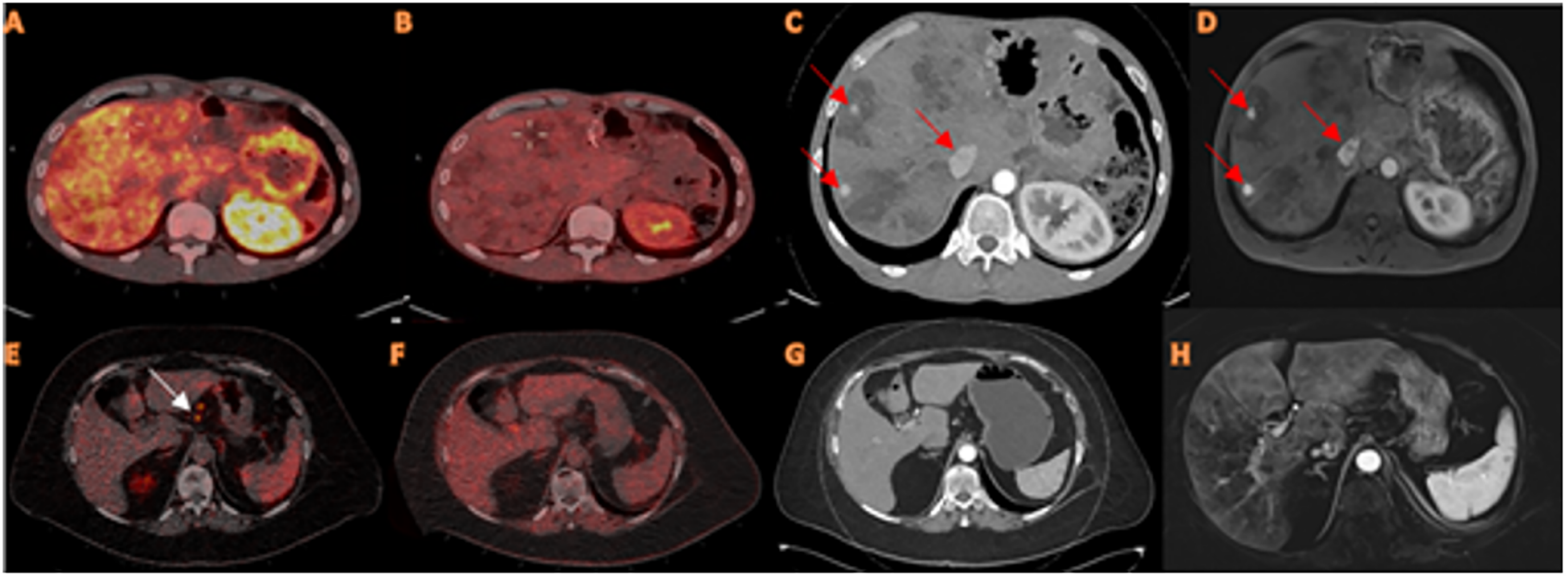
A 39-year-old man (DK-526) with MEN1 complicated by metastatic insulinoma to the liver. **(A, B)** 68Ga-DOTATATE PET/CT **(A)** and 18F-FDOPA PET/CT **(B)** did not identify uptake in the area of the focal enhancing lesions on CT/MRI. **(C, D)** CT **(C)** and MRI **(D)** show diffuse liver involvement with lesions ranging from 0.3 to 0.9 cm (red arrow). **(E–H)** Functional and anatomic abdominal imaging in a female with MEN1 (DK-143). **(E)** 68Ga-DOTATATE PET/CT demonstrates uptake of two metastatic lymph nodes near the gastric wall (white arrow), not seen on any other imaging (SUV maximum 35.5 and 31.5); white arrow. **(F–H)** 18F-FDOPA PET/CT **(F)**, computed tomography (CT) **(G)**, and magnetic resonance imaging (MRI) **(H)** failed to demonstrate the corresponding lymph nodes.

Liver: A total of 28 liver lesions were detected among 10 patients. Notably, in two patients, ^68^Ga-DOTATATE PET/CT localized three lesions that were not seen on CT/MRI. Overall, ^18^F-FDOPA PET/CT only identified 7.1% of the liver lesions (2/28; 95% CI: 0%–19.1%) with a SUVmax of 45 and 58, respectively. Interestingly, ^68^Ga-DOTATATE PET/CT and ^18^F-FDOPA PET/CT co-localized a liver metastasis with subtle ^68^Ga-DOTATATE uptake yet intense ^18^F-FDOPA PET/CT uptake in patient DK-59 (**Figure 2**). This patient had a urinary 5-HIAA that was three times the upper limit of normal, suggesting the functionality of this lesion. Liver lesions highly suspicious for metastasis were noted on CT/MRI in DK-526 with a known history of insulinoma. These lesions were not seen on ^18^F-FDOPA PET/CT and ^68^Ga-DOTATATE (**Figures 3A–D**).

Lymph nodes: ^18^F-FDOPA PET/CT identified 18.8% (6/32; 95% CI: 6.7%–33.3%) of lesions with a mean SUVmax of 12.8 ± 14.1 (range: 4.5–44.2) (**Figures 3E–H**).

Bone: Within our cohort, five patients were identified with bone lesions. On multidisciplinary review, all bone lesions were considered non-metastatic and consistent with other processes (e.g., arthritis, sclerosing bone cyst, and hemangioma).

Pituitary and parathyroid: Parathyroid lesions could not be comparatively analyzed given 100% of our patients have had a prior 2–3.5 gland parathyroidectomy and evaluation for residual or recurrent hyperparathyroidism requires specialized testing. Two pituitary lesions were identified on ^68^Ga-DOTATATE PET/CT with known corresponding MRI adenomas. Only physiologic pituitary uptake was identified on ^18^F-FDOPA PET/CT. Our study does not focus on pituitary or parathyroid imaging modalities as every patient did not undergo simultaneous pituitary or parathyroid imaging for confirmation (unlike the simultaneous chest, abdomen, and pelvic imaging).

### Biochemical correlations

An exploratory evaluation of biochemical variables of interest derived concurrently at the visit revealed that gastrin levels correlated with the number of lesions detected with ^68^Ga-DOTATATE PET/CT imaging (*r* = 0.5; *p* < 0.05) as well as lymph node metastases (*r* = 0.51; *p* < 0.042). No correlation of the number of lesions detected or lymph node metastases with ^68^Ga-DOTATATE PET/CT imaging was seen with serum glucose, chromogranin, or urinary 5-HIAA.

## Discussion

Tumor imaging is involved in every stage of GEP-NETs management for patients with MEN1. In combination with biochemical evaluations, imaging is essential for the identification of primary tumors and the detection of metastases (13). In addition, imaging assists in planning surgical resection and follow-up treatment for advanced or metastatic diseases. The current guidelines recommend abdominal MRI every 2–3 years in asymptomatic patients with MEN1 with negative baseline MRI and normal serum gastrin (6). CT is recommended for screening of lung NETs starting at age 20–25 years. SSTR scintigraphy PET/MRI or PET/CT is recommended for pre-operative planning or based on clinical implications (6). However, not all lesions demonstrate uniform SSTR expression highlighting the need for additional imaging approaches in selected cases. ^18^F-FDOPA PET/CT has not been investigated in patients with MEN1. In this cohort of patients with MEN1, ^18^F-FDOPA PET/CT demonstrated low sensitivity, with a detection rate of 17% of total tumor burden with minimal incremental value beyond ^68^Ga-DOTATATE PET/CT. Nearly all FDOPA-avid lesions were also identified on DOTATATE imaging, reinforcing the role of SSTR-based PET/CT as the primary functional imaging modality in this population.

^18^F-FDOPA PET/CT was evaluated because it interrogates a biologic pathway distinct from SSTR imaging, reflecting amine precursor uptake and decarboxylation in neuroendocrine cells. In MEN1, where tumors are frequently multifocal and heterogeneous, this raises the possibility that FDOPA may identify lesions with distinct metabolic or functional characteristics not fully captured by SSTR-based imaging. Although FDOPA showed limited sensitivity overall, selective uptake in a subset of lesions, including a case with discordant FDOPA-avid and weak DOTATATE uptake associated with elevated urine 5-HIAA, suggests that FDOPA may reflect underlying tumor heterogeneity and functional phenotype. One patient, DK-59, in our study had a large liver lesion with ^18^F-FDOPA PET/CT uptake and increased urinary 5-HIAA suggesting possible functionality. ^18^F-FDOPA uptake correlates with serotonin production and secretion, typically seen in midgut NETs but not in dpNETs, characteristic of MEN1 (14). Neuroendocrine cells with amine precursor uptake and decarboxylation are considered to have a higher 3,4-dihydroxy-L-phenylalanine (L-DOPA) decarboxylase activity and would be predicted to have better visualization on ^18^F-FDOPA PET/CT scans. Limited case reports have evaluated ^18^F-FDOPA PET/CT in patients with MEN1 with a pituitary prolactinoma (15), an 8-year old child with insulinoma (16), a pheochromocytoma (12), and a non-functional pNET (17). A retrospective study of 27 patients with NETs who were negative on conventional and SSTR scintigraphy showed a role for ^18^F-FDOPA PET/CT in the detection of primary NETs, especially tumors with a well-differentiated pattern and serotonin secretion (18). Prior studies in sporadic NETs have demonstrated that while SSTR-based imaging is superior for pancreatic NETs, FDOPA may have higher sensitivity in serotonin-secreting or midgut tumors. The limited performance observed in this MEN1 cohort is consistent with the predominance of dpNETs, which are less commonly associated with serotonin production. These findings further support the biological specificity of FDOPA uptake and its potential role in selected clinical contexts (14, 19).

Based on the findings of this study and the existing literature, the following clinical scenarios represent potential niche indications for ^18^F-FDOPA PET/CT in patients with MEN1. First, in our cohort, a serotonin-secreting hepatic lesion demonstrated avid FDOPA uptake in the absence of corresponding DOTATATE signal. This finding is consistent with published literature indicating that ^18^F-FDOPA PET/CT sensitivity for NETs was significantly associated with elevated serotonin or urinary 5-HIAA levels (83% vs. 20% in patients without serotonin elevation, *p* = 0.003) (18). Hence, in patients with MEN1 who have biochemical evidence of serotonin hypersecretion but negative or equivocal ^68^Ga-DOTATATE PET/CT, ^18^F-FDOPA PET/CT may localize the responsible lesion. Second, although pheochromocytoma is not a classic MEN1 manifestation, the discordant FDOPA-positive pheochromocytoma identified in our cohort illustrates a well-established advantage of this tracer. In patients with MEN1 with biochemical evidence of catecholamine excess and equivocal adrenal findings on SSTR imaging (where physiological adrenal cortical uptake can confound interpretation), ^18^F-FDOPA PET/CT offers superior lesion conspicuity due to the absence of normal adrenal gland uptake (20). Third, benign localized insulinomas typically lack SSTR overexpression, and ^18^F-FDOPA PET is identified as an alternative tracer being tested in this setting. In patients with MEN1 with functional syndromes (e.g., hypoglycemia and atypical hormonal elevations) where the responsible lesion is not identified on ^68^Ga-DOTATATE PET/CT, ^18^F-FDOPA PET/CT may provide complementary localization, particularly given the multiplicity of PNETs in MEN1 and the known limitations of SSTR imaging for certain functional tumor subtypes (5). A prospective study of 24 patients with ectopic ACTH syndrome demonstrated that ^18^F-DOPA PET/CT achieved 88% sensitivity (95% CI: 67%–97%), compared with 79% for ^68^Ga-DOTATATE (95% CI: 57%–92%) and 31% for Octreoscan (21). Thus, in patients with MEN1 presenting with Cushing syndrome from suspected ectopic ACTH production (e.g., thymic or bronchial carcinoid), dual-tracer functional imaging may optimize tumor localization. Finally, when curative-intent surgery is being considered in patients with MEN1 with metastatic disease, ^18^F-FDOPA PET/CT may detect additional lesions not seen on ^68^Ga-DOTATATE, particularly hepatic, peritoneal, and mesenteric nodal metastases. This incremental lesion detection could alter surgical planning.

In this study, 50% (8/16) of patients with MEN1 had ^18^F-FDOPA PET/CT uptake in MEN1-primary and metastatic sites with overlapping ^68^Ga-DOTATATE PET/CT uptake. There was only one lesion that was seen on ^18^F-FDOPA PET/CT that was not seen on ^68^Ga-DOTATATE PET/CT, which was in the adrenal and was histologically consistent with pheochromocytoma. Tumor multiplicity in distinct anatomical sites, the simultaneous presence of (often small) functional tumors, the heterogeneity of tumor presentation, and the unpredictable lesion malignant potential introduce complexity in the clinical management of patients with MEN1. In addition, tumor heterogeneity is a concept that has become increasingly recognized, especially in neuroendocrine neoplasms (22, 23). Reliance on radionucleotide tracer uptake as a surrogate for functionality is an emerging field in MEN1 (e.g., ^68^Ga-Exendin PET/CT as a sensitive predictor for insulinoma) (24) as well as molecular targets for therapeutic management (e.g., PRRT). These findings support efforts to better identify whether tumor heterogeneity in MEN1 can help predict malignant potential and/or inform future potential therapeutics. Ultimately, detection of a non-functional NET will be most useful when its malignant potential can be predicted to inform clinical decision-making. Our population included 75% of patients with Zollinger–Ellison syndrome, which has a prevalence of ~40% among patients with MEN1. While exploratory in nature, we found clear correlations between fasting serum gastrin levels and ^68^Ga-DOTATATE PET/CT uptake, which has been noted previously (25).

There are several limitations for this study. The sample size for our study is relatively small, reflecting the rarity of MEN1, which could lead to increased margins of error. Our study is also not a randomized controlled trial; patients were offered the opportunity to participate during their annual visits. In addition, histopathological confirmation was not available for all lesions, necessitating the use of a composite reference standard based on multidisciplinary review and longitudinal imaging. Because elements of SSTR-based imaging contributed to lesion adjudication, this study is not designed to compare the relative performance of ^18^F-FDOPA PET/CT vs. ^68^Ga-DOTATATE PET/CT. Thus, any conclusions regarding the relative efficacy of the two tracers for NETs are only suggestive. Finally, the cohort included a high proportion of patients with advanced diseases, which may limit generalizability to earlier-stage MEN1 populations.

## Conclusion

This study confirms that ^18^F-FDOPA PET/CT provides limited additional diagnostic value for GEP-NETs in patients with MEN1. However, the observed variability in ^18^F-FDOPA uptake underscores the functional heterogeneity of NETs with respect to amino acid transport and decarboxylase activity, which may have implications for understanding tumor biology and identifying subpopulations with distinct metabolic phenotypes. These findings support the continued use of SSTR-based imaging as the primary functional modality in MEN1, while suggesting that FDOPA may have a role in selected clinical scenarios that warrant further investigation.

## Data Availability

The datasets presented in this article are not readily available because data sharing will require evaluation of the request by the local Research Ethics Board and the signature of a data transfer agreement. Requests to access the datasets should be directed to the corresponding author(s).
